# Time-resolved role of P2X4 and P2X7 during CD8^+^ T cell activation

**DOI:** 10.3389/fimmu.2024.1258119

**Published:** 2024-02-15

**Authors:** Valerie J. Brock, Niels Christian Lory, Franziska Möckl, Melina Birus, Tobias Stähler, Lena-Marie Woelk, Michelle Jaeckstein, Joerg Heeren, Friedrich Koch-Nolte, Björn Rissiek, Hans-Willi Mittrücker, Andreas H. Guse, René Werner, Björn-Philipp Diercks

**Affiliations:** ^1^ The Calcium Signalling Group, Department of Biochemistry and Molecular Cell Biology, University Medical Centre Hamburg-Eppendorf, Hamburg, Germany; ^2^ Department of Immunology, University Medical Centre Hamburg-Eppendorf, Hamburg, Germany; ^3^ Department of Applied Medical Informatics, University Medical Centre Hamburg-Eppendorf, Hamburg, Germany; ^4^ Department of Computational Neuroscience, University Medical Centre Hamburg-Eppendorf, Hamburg, Germany; ^5^ Department of Biochemistry and Molecular Cell Biology, University Medical Centre Hamburg-Eppendorf, Hamburg, Germany; ^6^ Department of Neurology, University Medical Centre Hamburg-Eppendorf, Hamburg, Germany

**Keywords:** CD8 + T cells, Ca^2+^ imaging, activation, TCR stimulation, Ca^2+^ microdomains, NFAT, IFN-γ, proliferation

## Abstract

CD8^+^ T cells are a crucial part of the adaptive immune system, responsible for combating intracellular pathogens and tumor cells. The initial activation of T cells involves the formation of highly dynamic Ca^2+^ microdomains. Recently, purinergic signaling was shown to be involved in the formation of the initial Ca^2+^ microdomains in CD4^+^ T cells. In this study, the role of purinergic cation channels, particularly P2X4 and P2X7, in CD8^+^ T cell signaling from initial events to downstream responses was investigated, focusing on various aspects of T cell activation, including Ca^2+^ microdomains, global Ca^2+^ responses, NFAT-1 translocation, cytokine expression, and proliferation. While Ca^2+^ microdomain formation was significantly reduced in the first milliseconds to seconds in CD8^+^ T cells lacking P2X4 and P2X7 channels, global Ca^2+^ responses over minutes were comparable between wild-type (WT) and knockout cells. However, the onset velocity was reduced in P2X4-deficient cells, and P2X4, as well as P2X7-deficient cells, exhibited a delayed response to reach a certain level of free cytosolic Ca^2+^ concentration ([Ca^2+^]_i_). NFAT-1 translocation, a crucial transcription factor in T cell activation, was also impaired in CD8^+^ T cells lacking P2X4 and P2X7. In addition, the expression of IFN-γ, a major pro-inflammatory cytokine produced by activated CD8^+^ T cells, and Nur77, a negative regulator of T cell activation, was significantly reduced 18h post-stimulation in the knockout cells. In line, the proliferation of T cells after 3 days was also impaired in the absence of P2X4 and P2X7 channels. In summary, the study demonstrates that purinergic signaling through P2X4 and P2X7 enhances initial Ca^2+^ events during CD8^+^ T cell activation and plays a crucial role in regulating downstream responses, including NFAT-1 translocation, cytokine expression, and proliferation on multiple timescales. These findings suggest that targeting purinergic signaling pathways may offer potential therapeutic interventions.

## 1 Introduction

CD8^+^ T cells are part of the adaptive immune system and respond to intracellular pathogens such as viruses or bacteria. They recognize pathogen-derived antigen fragments presented on major histocompatibility complex (MHC) class I molecules with their T cell receptor (TCR) on the surface of infected cells. This triggers the release of a range of effector molecules, like perforin and granzymes, but also upregulation of Fas-ligand, ultimately resulting in direct apoptosis of the infected cells (1).

The initial activation of T cells can be visualized by the formation of highly dynamic Ca^2+^ microdomains. These initial signaling events are triggered by the most potent Ca^2+^-releasing second messenger, nicotinic acid adenine dinucleotide phosphate (NAADP) (2). NAADP binds to a small cytosolic binding protein, hematological and neurological expressed 1-like protein (HN1L)/Jupiter microtubule associated homolog 2 (JPT2) (3, 4) and in turn, evokes the release of Ca^2+^ from the endoplasmic reticulum (ER) through the ryanodine receptor type 1 (RYR1) in T cells (5, 6). This promotes the activation of stromal interaction molecules 1 and 2 (STIM1/2) and thus store-operated Ca^2+^ entry (SOCE) through ORAI1 channels (7), leading to an amplification of initial Ca^2+^ microdomains (6). Sustained Ca^2+^ microdomains result in the translocation of nuclear factor of activated T cells (NFAT) (8), which promotes the secretion of pro-inflammatory cytokines such as interleukin-17 (IL-17) (9) or interferon-γ (IFN-γ) (10). Interfering with the Ca^2+^ signaling cascade has potential therapeutic effects. For example, blocking SOCE by STX140 efficiently ameliorates the course of disease in a rat model of multiple sclerosis, where it prevents immune cells from infiltrating the central nervous system (11).

It is generally accepted that CD8^+^ T cells also play a central role in mediating anti-tumor immunity, by recognizing tumor-associated antigens presented on MHCI (12). Migration of CD8^+^ T cells into the tumor microenvironment (TME) correlates with improved prognosis in multiple cancer types (13, 14). The TME is especially rich in the potent immunosuppressive factor adenosine, which is generated by the degradation of extracellular adenosine triphosphate (eATP) through ectoenzymes (15). eATP is a pro-inflammatory signaling molecule that stimulates purinergic P2X receptors, an important mechanism regulating T cell activation, homing, and function (16). The two purinergic cation channels P2X4 and P2X7 were shown to play a role in T cell function (17) and amplify global Ca^2+^ signaling during T cell activation (18–20). Furthermore, in murine CD4^+^ T cells, P2X4 and P2X7 are essential to evoke initial Ca^2+^ microdomains in the first seconds after TCR stimulation (21). These signals were reduced by blocking the release of ATP through pannexin-1 or by degrading eATP using apyrase (21). However, the role of P2X4 and P2X7 on initial activation as well as the downstream effector function(s) in CD8^+^ T cells remains to be investigated.

Therefore, in the present study, we analyzed *P2rx4^-/-^* and *P2rx7^-/-^* CD8^+^ T cells across different timescales following TCR stimulation. We investigated the formation of initial Ca^2+^ microdomains during the first milliseconds – seconds, NFAT-1 translocation to the nucleus over 15 min, the expression of pro-inflammatory cytokines, like TNF-α and IFN-γ, and effector mediators like granzyme B up to 24 h post-TCR stimulation as well as the proliferation after 3 days. Taken together, our data suggest that purinergic signaling enhances initial Ca^2+^ events during CD8^+^ T cell activation and plays an essential role in fine-tuning the downstream activity of T cell activation.

## 2 Materials and methods

### 2.1 Reagents

Fluo4-AM and FuraRed-AM were purchased from Life Technologies and reconstituted in DMSO. Aliquots were stored at -20°C until assayed. Fura2-AM was obtained from Merck. Apyrase was dissolved in Ca^2+^ buffer in a final concentration of 100 U/mL, aliquoted, and stored at -20^°^C until used. Anti-mouse CD3 and anti-mouse CD28 monoclonal antibodies were obtained from BD Biosciences.

### 2.2 Animal models

*P2rx4^-/-^* (P2rx4tm1Rass; MGI (Mouse Genome Informatics):3665297) and *P2rx7^-/-^* mice (P2rx7tm1Gab; MGI:2386080) were backcrossed onto the BALB/c background as previously described (21) and analyzed together with WT BALB/c mice. They were bred at the animal facility of the University Medical Centre (UKE). The regulatory committee (Hamburger Behörde für Gesundheit und Verbraucherschutz, Veterinärwesen/Lebensmittelsicherheit, ORG 941 and 1115) approved the experiments, which included tissues of animal origin.

### 2.3 Isolation of primary murine CD8^+^ T cells

Spleens and lymph nodes from WT or knockout BALB/C mice were passed through a 40 µm strainer and erythrocytes were lysed in ammonium-chloride-potassium (ACK) buffer (4.3 g ammonium chloride, 0.5 g KHCO_3_, 0.0186 g Na_2_-EDTA in 400 mL H_2_O, pH 7.2–7.4) for 3-5 min. CD8^+^ T cells were separated from other cells using a negative selection kit (EasySep Mouse CD8^+^ T Cell Enrichment Kit, STEMCELL Technologies Inc.). Cell purity of 97-98% was validated by FACS.

### 2.4 Imaging initial Ca^2+^ microdomains in primary murine CD8^+^ T cells

CD8^+^ T cells, isolated on the day of the measurement, were loaded with the Ca^2+^ dyes Fluo4 (10 µM) and FuraRed (20 µM), and Ca^2+^ imaging was carried out as described in detail by Diercks et al., 2019 (22). Cells were imaged in Ca^2+^ buffer [140 mM NaCl, 5 mM KCl, 1 mM MgSO_4_, 1 mM CaCl_2_, 20 mM Hepes (pH 7.4), 1 mM NaH_2_PO_4_, 5 mM glucose] for three minutes on coverslips coated with 5 µl BSA (5 mg/mL) and 5 µl Poly-L-Lysine (0.1 mg/mL) to increase adhesion of the cells. To remove extracellular ATP, 10 U/mL apyrase was added to the cells 3 minutes prior to imaging. T cells were stimulated with anti-CD3/anti-CD28 coated beads after the first minute of measurement. Small, compact connected pixel sets with high [Ca^2+^]_i_ values were defined as Ca^2+^ microdomains. The signals occurring after stimulation were detected in an automated MATLAB script (22). The location and time of the bead contact of all individual cells were normalized and an average value for all cells of a condition was formed and displayed in a dartboard projection for a specific time window, e.g. the first second after the bead contact.

### 2.5 Global Ca^2+^ imaging in primary murine CD8^+^ T cells

CD8^+^ T cells, which were isolated on the day of the experiment, were loaded with 4 µM Fura2-AM for 35 min at 37°C. Global Ca^2+^ imaging was performed as described by Weiß and colleagues, 2023 (8). Cells were imaged in Ca^2+^ buffer [140 mM NaCl, 5 mM KCl, 1 mM MgSO_4_, 1 mM CaCl_2_, 20 mM Hepes (pH 7.4), 1 mM NaH_2_PO_4_, 5 mM glucose] for 8 min on coverslips coated with 5 µL BSA (5 mg/mL) and 5 µL Poly-L-Lysine (0.1 mg/mL). Monoclonal Anti-CD3 antibody (1 µg/mL) was used to stimulate the cells one minute after the measurement started. Images were acquired in 16-bit mode with Volocity software (PerkinElmer). FIJI software (version 1.53f) was used for post-processing like background correction, splitting of the fluorescence channels, and selection of the regions of interest (ROI).

### 2.6 Super-resolution NFAT1 translocation

The preparation of slides was performed as described in 2.4. Next, CD8^+^ T cells were seeded on the slides, fixed, and permeabilized. Cells were treated with biotin anti-CD3 (553060, BD Biosciences)/anti-CD28 (553296, BD Biosciences) coated transparent streptavidin beads (TP190902KOM5, PolyAn) for either 7.5 min or 15 min. Overnight incubation was performed with 10% (v/v) fetal calf serum (FCS) to block non-specific binding sites. Primary antibody staining to visualize NFAT-1 (rabbit anti-mouse NFAT-1, 1:200 (5861S, Cell Signaling Technology) was applied for 1 hour at room temperature. Furthermore, the cells were washed four times with 3% (v/v) FCS to remove the primary antibody. The samples were again stained for 1 hour at room temperature with a secondary antibody (anti-rabbit Alexa Fluor 488, 1:400 (A21206, Life technologies). The nucleus was labeled by DAPI staining (62247, ThermoFisher, 1:1000) for 10 min at room temperature. The glass slides were mounted overnight with Abberior Mount Solid (Abberior). Image acquisition was carried out using a high-resolution spinning disk (Visitron) containing a CSU-W1 SoRa Optic (2,8x, Yokogawa), a 100x magnification objective (Zeiss) and a sCMOS camera (Orca-Flash 4.0, C13440-20CU Hamamatsu). The ratio of the nuclear NFAT-1 signal divided by the NFAT-1 signal in the cytoplasm was calculated as a percentage to analyze the translocation of NFAT-1. Spots were marked by pixel-based segmentation using the trainable weka (Waikato Environment for Knowledge Analysis) segmentation plugin on Fiji (version 1.53f). The area (sum of detected spots) of all NFAT-1 spots in the nucleus and cytoplasm was calculated using a MATLAB (version R2018b) script (8). To distinguish between the nucleus and the cytoplasm DAPI staining was used. Our results were normalized using the dynamic range (baseline to maximum response) determined by the 15 min bead stimulation of our NFAT-1 translocation assay, which included a baseline of 39% and a maximum response of approximately 70%, as noted.

### 2.7 Expression analysis by flow cytometry

Spleen cells were isolated and erythrocytes were depleted with ACK buffer as described above. Spleen cells were incubated in 500 μl of Iscove’s modified Dulbecco’s medium (IMDM). Fetal calf serum, glutamine, gentamicin, and 2-mercaptoethanol were added to the medium. Stimulation of the cells was performed using anti-CD3ϵ mAb (1 μg/mL; clone 145-2C11, BioLegend, San Diego, CA). For some assays, anti-CD28 mAb (1 μg/mL; clone 37.51, BioLegend) was added, as indicated in the figure legends. Brefeldin A (Sigma-Aldrich) was added during the last 4 hours of culture for cytokine production assays. For extracellular antibody staining, cells were incubated in PBS with 1% rat serum and anti–Fc receptor mAb (10 μg/ml; clone 2.4G2, BioXCell, West Lebanon, NH) for extracellular antibody staining. A fixable dead cell stain (Alexa Flour 750 carboxylic acid, succinimidyl ester, Invitrogen, Eugene, OR), V450-conjugated anti-CD8 mAb (clone 53-6.7, BD Biosciences) and BV605-conjugated anti-CD69 mAb (clone H1.2F3, BioLegend) were then added to the cells for 20 minutes on ice. Intracellular antibody staining was performed using the Foxp3/Transcription Factor Staining Buffer Set (Invitrogen) following the manufacturer’s protocol. Cells were stained with PE-conjugated anti-NUR77 mAb (clone 12.14, Invitrogen), PE-Cy7-conjugated anti-IRF4 mAb (clone 3E4, eBioscience, San Diego, CA), FITC-conjugated anti-Granzyme B mAb (clone GB11, BioLegend), AF647-conjugated IFN-γ mAb (clone XMG1.2; eBioscience), and FITC-conjugated anti-TNF-α mAb (clone MP6-XT22, BioLegend). A FACSCelesta flow cytometer (BD Biosciences, Franklin Lakes, NJ) and FlowJo software (Tree Star, Ashland, OR) were used for cell analysis.

To evaluate proliferation, spleen cells were treated with carboxyfluorescein diacetate succinimidyl ester (CFSE, 5 μM; Invitrogen) for 10 minutes at room temperature and then washed twice. They were stimulated with anti-CD3ϵ mAb (1 μg/mL; clone 145-2C11, BioLegend) and anti-CD28 mAb (1 μg/mL; clone 37.51, BioLegend) in supplemented IMDM. After 3 days, cells were stained with V450-conjugated anti-CD8 mAb (clone 53-6.7, BD Biosciences) and a fixable dead cell stain (Alexa Flour 750 carboxylic acid, succinimidyl ester, Invitrogen). Cell analysis was performed using FACS. Proliferation is given as the Expansion index (EI) indicating the overall expansion of the culture (23), which was calculated with the FlowJo software.

### 2.8 Statistics

All data are presented as the mean ± SEM of independent experiments, which were performed as a minimum in triplicate. Data were analyzed by MATLAB software (MathWorks), Prism 9 (GraphPad Software), and Excel (Microsoft). Data were tested for normality using the Kolmogorov-Smirnov test with Dallal-Wilkinson-Lilliefors P-value. Normal distributed data sets were compared by using ordinary one-way analysis of variance (ANOVA) and Tukey’s multiple comparisons test. Data sets lacking normal distribution were analyzed using Kruskal-Wallis tests and Dunnett’s multiple comparisons tests. Data from flow cytometry experiments were compared with two-way ANOVA and Tukey’s multiple comparisons test. A p value below 0.05 was considered to be significant.

## 3 Results

### 3.1 P2X4 and P2X7 are involved in the formation of initial Ca^2+^ microdomains in primary CD8^+^ T cells during the first milliseconds to seconds after stimulation

**Figure 1:**
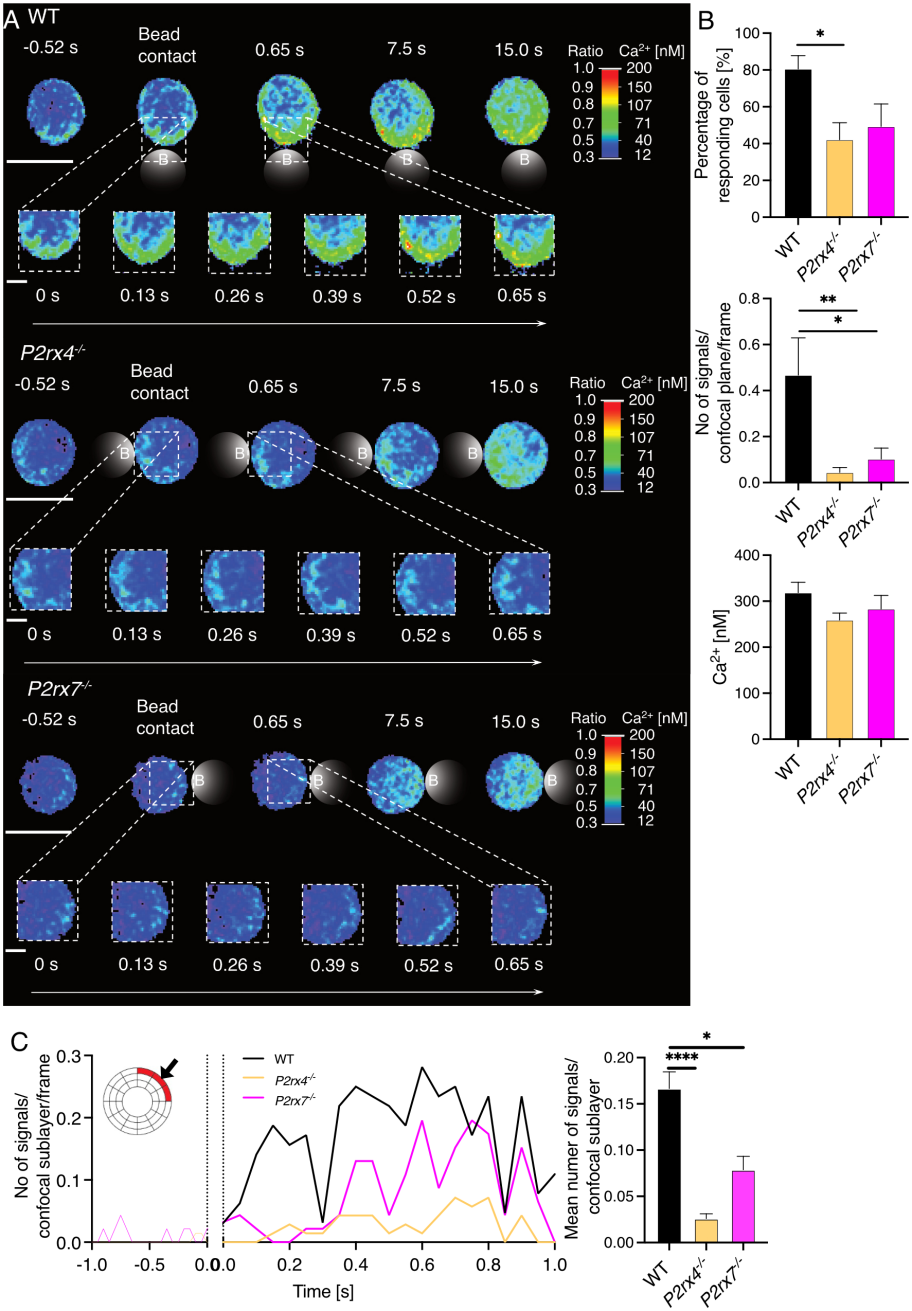
P2X4 and P2X7 influence the formation of Ca^2+^ microdomains in the first seconds after TCR stimulation. Initial Ca^2+^ microdomain imaging of CD8^+^ T cells of WT, *P2rx4* ^-/-^ and *P2rx7* ^-/-^ mice was performed with the Ca^2+^ indicators Fluo4 and FuraRed using anti-CD3/anti-CD28 coated beads. For each condition, 7 mice were used. **(A)** A representative cell of WT, *P2rx4* ^-/-^ and *P2rx7* ^-/-^ is shown for a time window of -0.52 s to 15 s after bead engagement (scalebar 5 µm) and a magnified region of the bead contact site from 0 s - 0.65 s (scalebar 1 μm). B-C) Data are mean ± SEM, WT n = 32 cells, *P2rx4* ^-/-^ n = 35 cells, *P2rx7* ^-/-^ n = 23 cells. N = 7 per group. Results were analyzed using the Kruskal-Wallis test and Dunnett’s multiple comparisons test (*p < 0.05; **p < 0.01, ****p < 0.0001). **(B)** Quantification of the initial Ca^2+^ microdomains in CD8^+^ T cells 15 s after bead engagement. The percentage of responding cells (upper panel), the number of Ca^2+^ microdomains per frame for whole cells (confocal plane, middle panel) and the average Ca^2+^ amplitude of these signals are shown (lower panel). **(C)** Analysis of the initial Ca^2+^ microdomains in a time window -1 s to 1s after bead engagement for contact site sublayer of a dartboard projection (as indicated in red, left panel) and quantification of these signals (right panel).

**Figure 2:**
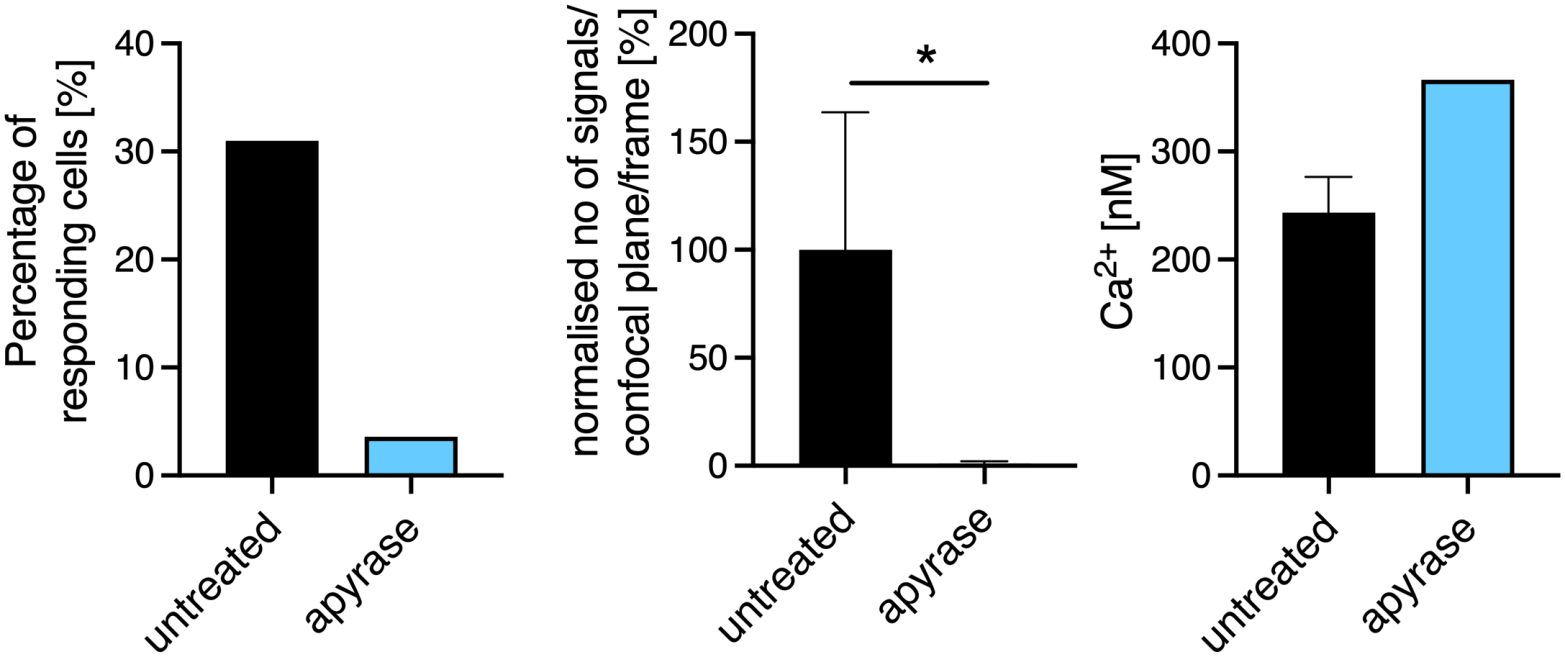
Extracellular ATP (ATPe) influences the formation of Ca^2+^ microdomains in the first seconds after TCR stimulation. Initial Ca^2+^ microdomain imaging of CD8^+^ T cells of WT with and without addition of apyrase to the Ca^2+^ measurement buffer was performed with the Ca^2+^ indicators Fluo4 and FuraRed using anti-CD3/anti-CD28 coated beads. Bar charts show the percentage of responding cells (left panel), the normalized number of Ca^2+^ microdomains per frame for whole cells (per confocal plane, middle panel), the average Ca^2+^ amplitude of these signals are shown (right panel) 15s after bead engagement. Data are mean ± SEM, untreated n = 20 cells, apyrase n= 21 cells from 7 independent experiments. Results were analyzed using the Mann-Whitney test (*p < 0.05).

CD8^+^ T cells were freshly isolated from wild-type (WT), *P2rx4^-/-^* and *P2rx7^-/-^* mice on a BALB/c background (24) and stimulated with anti-CD3/anti-CD28–coated beads in order to imitate an immune synapse. Initial Ca^2+^ microdomains were analyzed 0.5 s prior to and up to 15 s after bead contact using high-resolution Ca^2+^ live-cell imaging (5, 6) (**Figure 1A**). Previously, such Ca^2+^ microdomains were defined by a signal area of 0.216± 0.004 µm^2^ (mean ± SEM) and an average amplitude of 325 ± 11 nM (mean ± SEM) (5, 6, 25). Directly after bead contact in the first 500 ms, in CD8^+^ WT T cells (**Figure 1A**, top) single local Ca^2+^ microdomains at the bead contact site were detectable, spreading through the whole cell within 15 s. In contrast, in CD8^+^ T cells from *P2rx4^-/-^* and *P2rx7^-/-^* mice (**Figure 1A**, middle and bottom) these initial Ca^2+^ microdomains directly after bead contact were absent. In 80% of CD8^+^ WT cells, initial Ca^2+^ microdomains were detected, with a mean amplitude of 319 ± 22 nM (mean ± SEM, n=32) and 0.46 signals/confocal plane/frame, which corresponds to approximately 18 Ca^2+^ microdomains/confocal plane/second (**Figure 1B**). Only 42% of *P2rx4^-/-^* cells and 49% of *P2rx7^-/-^* cells responded with initial Ca^2+^ microdomains, with significantly decreased numbers of Ca^2+^ signals/confocal plane/frame of 0.04 (*P2rx4^-/-^*; n=35) and 0.1 (*P2rx7^-/-^*; n = 23*)*, and mean Ca^2+^ amplitudes of 259 ± 15 nM (mean ± SEM, n = 16) and 283 ± 29 nM (mean ± SEM, n = 10) (**Figure 1B**). Already in the first milliseconds after bead contact the numbers of initial Ca^2+^ microdomains directly at the artificial immune synapse was significantly reduced (**Figure 1C**). While cells lacking the P2X4 channel showed nearly no increase of Ca^2+^ microdomains in the first milliseconds after TCR stimulation, a slight increase was observed for cells lacking the P2X7 channel. This slight increase was lower and delayed compared to the Ca^2+^ response of WT T cells at the artificial immune synapse (**Figure 1C**). Furthermore, if the extracellular ATP (eATP) was hydrolysed by recombinant apyrase the number of initial Ca^2+^ microdomains was significantly reduced in WT CD8^+^ T cells by 99% (**Figure 2**). Only in one of the 21 T cells treated with extracellular apyrase Ca^2+^ microdomains could be detected, while 30% of the untreated cells responded (**Figure 2**). Taken together, the initial Ca^2+^ response milliseconds after T cell stimulation in CD8^+^ T cells is promoted by eATP stimulating the purinergic cation channels P2X4 and P2X7.

### 3.2 Global Ca^2+^ signals in the first minutes are impaired in primary CD8^+^ *P2rx4^-/-^* and *P2rx7^-/-^* T cells

**Figure 3:**
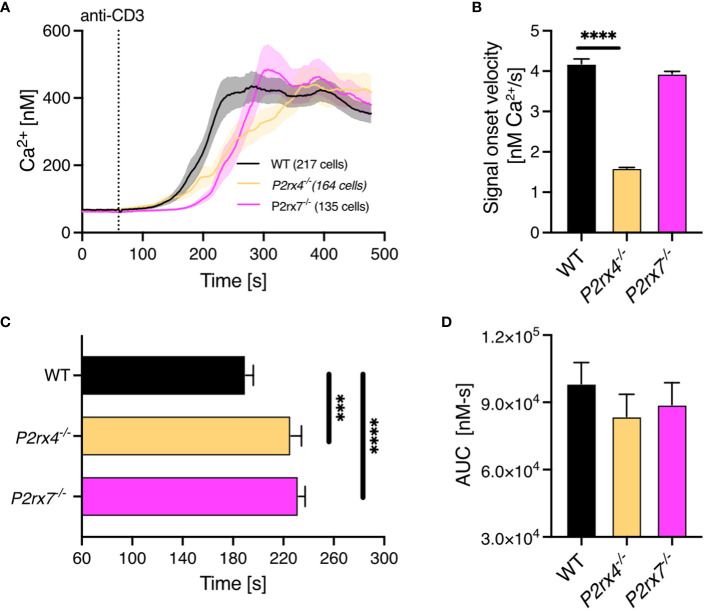
Impaired global Ca^2+^ response in CD8^+^ T cells from *P2rx4* ^-/-^ and *P2rx7* ^-/-^ mice. Ca^2+^ imaging of CD8^+^ T cells of WT, *P2rx4* ^-/-^ and *P2rx7* ^-/-^ mice was performed with the Ca^2+^ indicator Fura-2 for 8 min. **(A)** Cells were stimulated with monoclonal antibodies against CD3 (10µg/mL) after the first min of measurements. For each condition, 3 mice were used (N = 3 per group). Data are mean ± SEM, WT n = 217 cells, *P2rx4* ^-/-^ n = 164 cells, *P2rx7* ^-/-^ n = 135 cells. Results were analyzed using Kruskal-Wallis test and Dunnett’s multiple comparisons test (***p < 0.001, ****p < 0.0001). The data were quantified by analyzing the signal onset velocity **(B)**, the time to reach 200 nM Ca^2+^ **(C)** and the area under the curve **(D)**.

The transition of early, spatiotemporally restricted Ca^2+^ microdomains into a global Ca^2+^ response indicates regular T cell function. Thus, we analyzed global Ca^2+^ signals in the first 8 mins after TCR stimulation with soluble antibodies against CD3. The overall Ca^2+^ response in WT (n = 217) compared to *P2rx4^-/-^* (n = 164) and *P2rx7^-/-^* (n = 135) CD8^+^ T cells appeared similar (**Figure 3A**). However, compared to WT T cells the signal onset velocity was 2.6-fold reduced in *P2rx4^-/-^* T cells while there was no difference in *P2rx7^-/-^* CD8^+^ T cells (**Figure 3B**). In contrast the time to reach a 200 nM threshold was significantly delayed in *P2rx4^-/-^* and *P2rx7^-/-^* T cells (**Figure 3C**). T cells lacking either the P2X4 or P2X7 channel reached the threshold after 226 ± 8 s (mean ± SEM, n = 135) or 234 ± 5 s (mean ± SEM, n = 164) compared to WT cells reaching the threshold already after 119 ± 6 s (mean ± SEM, n = 217). Furthermore, there was no significant difference in the area under the curve (AUC) (**Figure 3D**). In summary, the global Ca^2+^ response during the first 8 mins after TCR stimulation is altered in CD8^+^ T cells from *P2rx4^-/-^* and *P2rx7^-/-^* mice but in different aspects.

### 3.3 NFAT-1 translocation after 15 minutes is reduced in primary CD8^+^ *P2rx4^-/-^* and *P2rx7^-/-^* T cells

**Figure 4:**
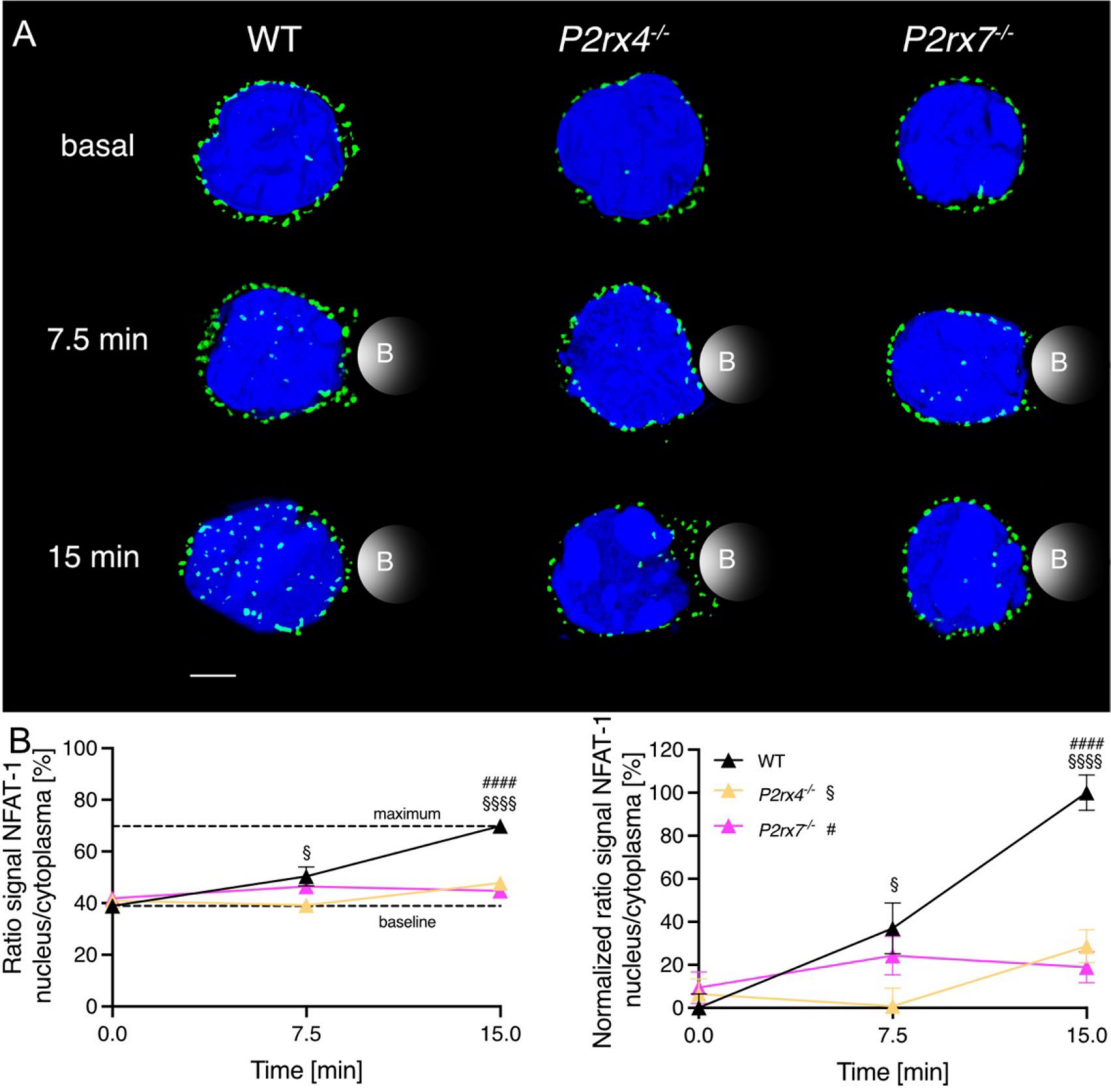
The nuclear translocation of NFAT-1 is influenced by P2X4 and P2X7 minutes after TCR stimulation. CD8^+^ T cells of WT, *P2rx4* ^-/-^ and *P2rx7* ^-/-^ mice were activated with anti-CD3/anti-CD28 coated beads for 7.5 min and 15 min and NFAT-1 protein and the nucleus were stained. Three spleens per condition were used. **(A)** A representative cell for WT, *P2rx4* ^-/-^ and *P2rx7* ^-/-^ without stimulation (basal), and after 7.5 min and 15 min stimulation are shown. NFAT-1 is presented in green, the nucleus in blue. Bead contact is indicated schematically. Scalebar 2 µm. **(B)** The translocation of NFAT-1 is quantified by the ratio of the signal of NFAT-1 in the nucleus/cytoplasm (right panel). Approximation of the dynamic range using the maximal response, which was obtained with 15 min of bead stimulation in WT T cells and data were normalized to this dynamic range (left panel). Data are means ± SEM. Basal WT n = 61 cells, *P2rx4* ^-/-^ n = 38 cells and *P2rx7* ^-/-^ n = 56 cells. 7.5 min WT n = 29 cells, *P2rx4* ^-/-^ n = 29 cells and *P2rx7* ^-/-^ n = 33 cells. 15 min WT n = 35 cells, *P2rx4* ^-/-^ n = 36 cells and *P2rx7* ^-/-^ n = 24 cells. Data were compared using ordinary one-way ANOVA with Dunnett’s multiple comparisons test (P-values § for *P2rx4* ^-/-^, # for *P2rx7* ^-/-^, p §/# < 0.05, and p §§§§/#### < 0.0001).

NFAT-1 is one of the major transcription factors for T cell activation. The translocation of NFAT-1 is regulated by Ca^2+^ and the phosphatase calcineurin (26). NFAT-1 is present in naïve CD8^+^ T cells and its dephosphorylation is detectable already 15 min after stimulation with ionomycin (27). Hence, we analyzed the translocation of NFAT-1 in primary murine CD8^+^ WT, *P2rx4^-/-^* and *P2rx7^-/-^* T cells after 7.5 and 15 min of bead stimulation. Unstimulated T cells from WT (n = 61) or knockout mice (*P2rx4^-/-^* n = 38; *P2rx7^-/-^* n = 56) showed an equal distribution of NFAT-1 protein (indicated in green) outside the nucleus (indicated in blue) in the cytoplasm (**Figure 4A**, upper row). There was no difference in the percentage of nuclear NFAT-1 signal in CD8^+^ T cells without stimulation (basal). After 7.5 min bead stimulation, moderate NFAT-1 translocation of 37 ± 12% (mean ± SEM, n = 29) was visible in WT CD8^+^ T cells but only marginally detected in *P2rx4^-/-^* with 0.8 ± 8% (mean ± SEM, n = 29) and *P2rx7^-/-^* T cells with 24 ± 9% (mean ± SEM, n = 33) (**Figure 4B** right panel). The differences between WT and *P2rx4^-/-^* or *P2rx7^-/-^* T cells increased to a highly significant level after 15min. In comparison to the WT (normalized ratio signal of 100 ± 8%, n = 35), only 29 ± 8% (mean ± SEM, n = 36) and 19 ± 7% (mean ± SEM, n = 24) of nuclear NFAT-1 protein signal was detected in *P2rx4^-/-^* and *P2rx7^-/-^* T cells (**Figure 4B** right panel). The separated individual channels, as well as brightfield images with beads, are shown in **Supplementary Figure 1**. On the other hand, if proximal TCR signaling is bypassed with the addition of thapsigargin (positive control) a comparable NFAT-1 translocation in all genotypes was induced (**Supplementary Figure 2**). These results indicate the involvement of P2X4 and P2X7 in the signal transduction following TCR stimulation of CD8^+^ T cells from early Ca^2+^ signals to the transcriptional response.

### 3.4 P2X4 and P2X7 are involved in cytokine and activation marker expression at 18–24 hours and proliferation at days in primary CD8^+^ T cells

**Figure 5:**
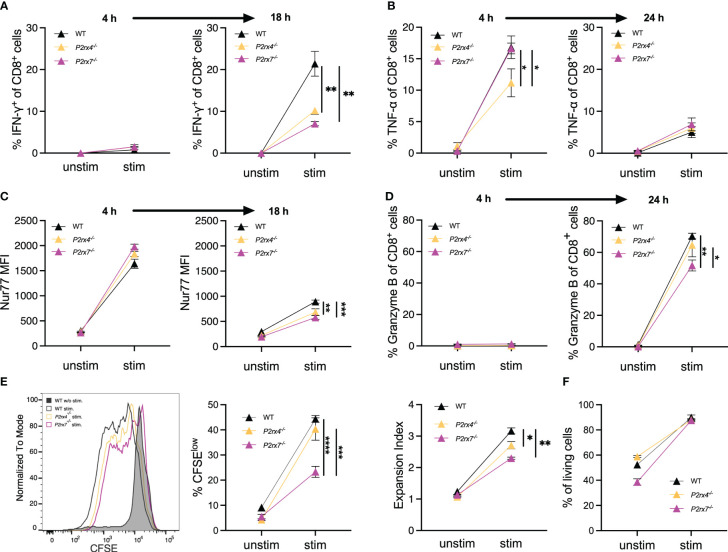
P2X4 and P2X7 are involved in the expression of cytokines and activation markers as well as the induction of proliferation. **(A–D)** Spleen cells from WT, *P2rx4* ^-/-^ and *P2rx7* ^-/-^ mice were incubated with and without anti-CD3 mAb and anti-CD28 mAb **(A, C)** or only anti-CD3 mAB **(B, D)**. After 4 h and 18 h or 24h, the expression of IFN-γ **(A)**, TNF-α **(B)**, Nur77 **(C)** and granzyme B **(D)** was analyzed by FACS given as % of CD8^+^ T cells **(A, B, D)** or mean fluorescence intensity (MFI) of antibody staining **(C)**. **(E)** Spleen cells from WT, *P2rx4* ^-/-^ and *P2rx7* ^-/-^ mice were labeled with CFSE and incubated with and without anti-CD3 mAb and anti-CD28 mAb. Representative results for CFSE staining of CD8^+^ T cells is shown in the histogram. The strength of proliferation is given as % CFSE_low_ and as expansion index which takes into account the number of divisions. **(F)** Survival of cells was estimated by % of living cells after culture for 72h with and without stimulation. Mean ± SEM, N = 3 per group. **(F)** Results were analyzed with two-way ANOVA and Tukey’s post-test (*p < 0.05; **p < 0.01; ***p < 0.001; ****p < 0.0001).

Previously, it was shown that the key regulator of IFN-γ production is NFAT-1 (28). The expression of IFN-γ occurs rapidly after T cell stimulation and is linked to several cell functions, like cytotoxicity (29, 30). Since NFAT-1 translocation is reduced in CD8^+^ T cells from *P2rx4^-/-^* and *P2rx7^-/-^* mice (**Figure 4**) and NFAT-1 is the major regulator of IFN-γ production (28), we analyzed the expression of IFN-γ after stimulation in the WT and both knockout cells. After 4 h, only 1-2% of the CD8^+^ T cells also express IFN-γ, with a little higher expression in *P2rx4^-/-^* and *P2rx7^-/-^* T cells (**Figure 5A**). An increase in T cells expressing IFN-γ was observed after 18 h for WT and *P2rx4^-/-^* and *P2rx7^-/-^* mice. At this time point, a significantly lower percentage of *P2rx4^-/-^* and *P2rx7^-/-^* T cells of 10 ± 1% (mean ± SEM, N = 3) and 7 ± 1% (mean ± SEM, N = 3) express IFN-γ compared to 21 ± 2% (mean ± SEM, N = 3) of WT T cells (**Figure 5A**; **Supplementary Figure 4**). Another important proinflammatory cytokine whose expression is regulated by NFAT-1 is tumor necrosis factor alpha (TNF-α) (31). Analyzing TNF-α positive T cells with and without stimulation after 4h revealed only a significant reduction in the percentage of *P2rx4* ^-/-^ T cells (11 ± 2%; mean ± SEM, N = 3), while the percentage of *P2rx7* ^-/-^ (17 ± 1%; mean ± SEM, N = 3) and WT T cells (17 ± 2%; mean ± SEM, N = 3) are almost identical (**Figure 5B**). Interestingly, after 24h the induced expression of TNF-α was generally much weaker in CD8^+^ T cells from mice of all genotypes (**Figure 5B**; **Supplementary Figure 4**). Critical for the effector function of CD8^+^ T cells is the expression and release of granzyme B (32). Without stimulation and after stimulation for 4h, there was hardly any expression of granzyme B in CD8^+^ T cells from mice of all genotypes (between 0-1%) but granzyme B was strongly induced after stimulation for 24h (**Figure 5D**). For granzyme B, we also observed weaker induction in CD8^+^ T cells from *P2rx4* ^-/-^ (65 ± 8%; mean ± SEM, N = 3) and particularly from *P2rx7* ^-/-^ (52 ± 4%; mean ± SEM, N = 3) mice compared to WT (70 ± 2%; mean ± SEM, N = 3) (**Figure 5D**; **Supplementary Figure 4**). To further assess the effects of P2X4 and P2X7 on downstream signaling in CD8^+^ T cells, the expression of Nur77, IRF4, and CD69 were measured after 4h and 18h of TCR stimulation. The transcription factors Nur77 and IRF4 are expressed immediately after TCR stimulation and their expression has been associated with the strength of TCR signal (33, 34). CD69 is an early surface marker of activated T cells. Following TCR stimulation, we observed the induction of all three proteins in WT CD8^+^ T cells (**Figure 5C**, **Supplementary Figures 3A, B**). Compared to WT CD8^+^ T cells, *P2rx4* ^-/-^ and *P2rx7* ^-/-^ T cells showed similar expression of IRF4 (**Supplementary Figures 3B, 4**). Expression of Nur77 and CD69 was reduced in *P2rx4* ^-/-^ and *P2rx7* ^-/-^ T cells, but only for Nur77 in *P2rx4* ^-/-^ and *P2rx7* ^-/-^ T cells reduction reached a level of significance. Compared to the WT T cells (893 ± 30 mean fluorescence intensity (MFI); mean ± SEM, N = 3), T cells lacking P2X4 (684 ± 68 MFI; mean ± SEM, N = 3) or P2X7 (579 ± 38 MFI; mean ± SEM, N = 3) showed a significant reduced Nur77 expression 18h after stimulation. Representative histograms and plots of all flow cytometry stainings are shown in **Supplementary Figure 4**. Next, the proliferation of cells was assessed by dilution of CFSE staining 3 days after incubation with anti-CD3 mAb (**Figure 5E**, **Supplementary Figure 5**). Compared to WT CD8^+^ T cells, CD8^+^ T cells of *P2rx4^-/-^* mice showed slightly lower and of *P2rx7^-/-^* mice significant lower frequencies of CFSE^low^ cells after stimulation. When the expansion index (EI) was calculated, which takes into account the number of divisions, both *P2rx4* ^-/-^ and *P2rx7* ^-/-^ CD8^+^ T cells showed significantly reduced proliferation which was again slightly more pronounced for *P2rx7* ^-/-^ T cells (EI after stimulation: WT 3.1 ± 0.10, N = 3), for *P2rx4* ^-/-^ of 2.7 ± 0.12, N = 3 and *P2rx7* ^-/-^ of 2.3 ± 0.04, N = 3). To control if these effects were not due to different cell survival the percentage of living cells was measured (**Figure 5F**). No difference between T cells from the different genotypes after stimulation for 72h was observed (**Figure 5F**). As a control, T cells were stimulated with Phorbol 12-myristate 13-acetate (PMA) and the Ca^2+^ ionophore ionomycin. With this stimulus, which bypasses proximal TCR signaling, there was similar upregulation of Nur77 and CD69 in CD8^+^ T cells from all genotypes (**Supplementary Figure 5**). In conclusion, these results indicate that P2X4 and P2X7 differentially affect the expression of TCR-induced proteins and the proliferation of CD8^+^ T cells.

**Figure 6:**
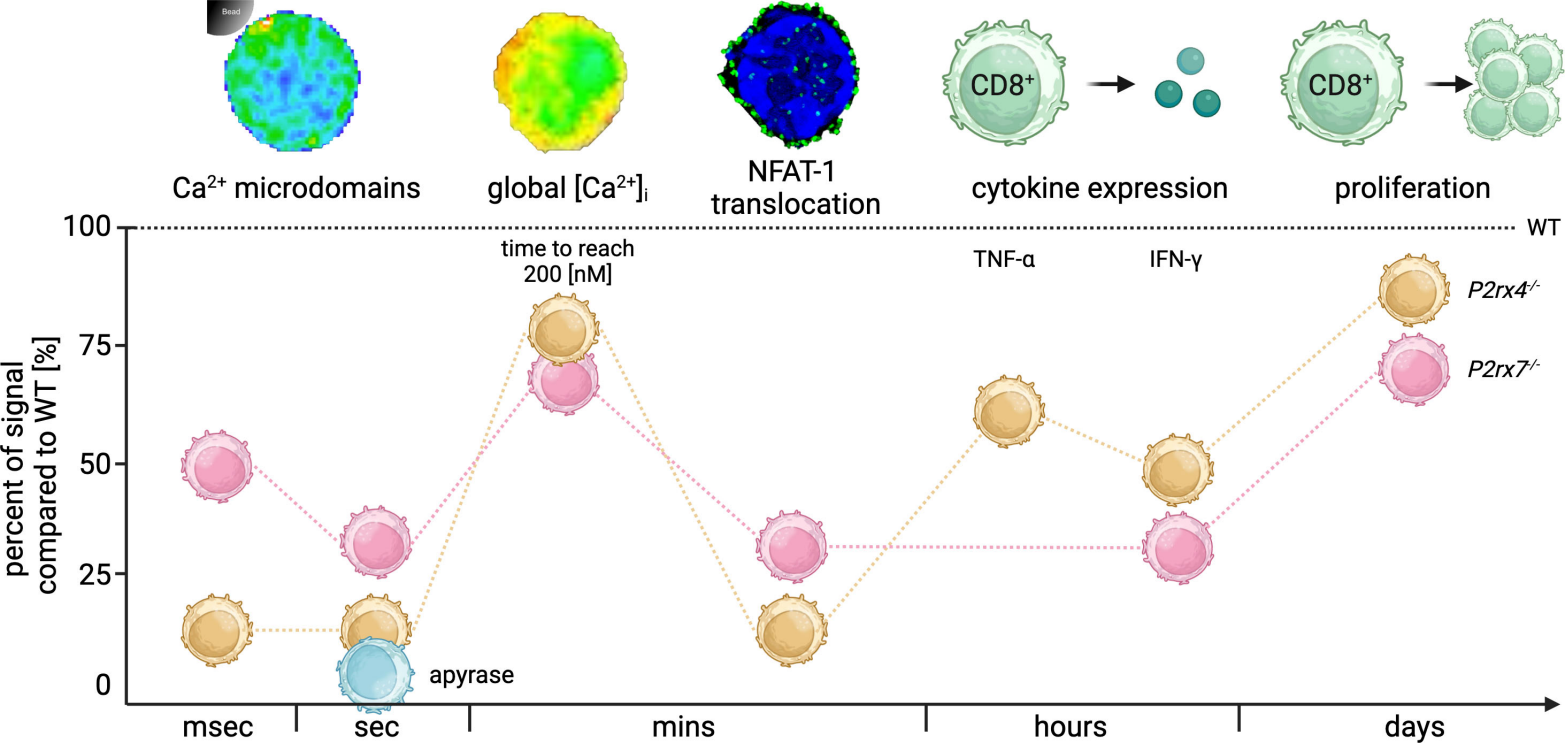
Summary of the time-resolved role of P2X4 and P2X7 during CD8^+^ T cell activation. Summary of the significant signal reduction in CD8^+^ T cells from *P2rx4^-/-^* and *P2rx7^-/-^* compared to the WT (dashed line) in milliseconds (msec), seconds (sec), minutes (mins), hours and days after TCR stimulation. In the first msec Ca^2+^ microdomains were reduced in *P2rx4^-/-^* and *P2rx7^-/-^* T cells by 85% and 54%. Seconds after stimulation the Ca^2+^ microdomains were reduced by 91% and 78%. Moreover, the hydrolysis of eATP by the addition of apyrase (blue) reduces the number of Ca^2+^ microdomains by 99% in the first 15 seconds in WT CD8^+^ T cells. The global Ca^2+^ response in the first mins after stimulation was delayed and the cells lacking either P2X4 or P2X7 showed only 16% or 19% of the time to reach 200 nM Ca^2+^ compared to the WT. The translocation of the nuclear factor NFAT-1 was decreased in *P2rx4^-/-^* and *P2rx7^-/-^* T cells by 71% and 81% after mins, whereas cytokine expression of IFN-γ was decreased by 52% and 67%. Only in CD8^+^ T cells from *P2rx4* ^-/-^ mice, the number of TNF-α expressing cells was significantly reduced by 33%. Furthermore, the proliferation of the cells was affected by a reduction of 15% and 25% compared to WT cells. (Created with BioRender.com).

Consistently, the results from the current study demonstrate the influence of the purinergic cation channels P2X4 and P2X7 on CD8^+^ T cell activation across timescales, starting with initial signals in the first milliseconds after TCR stimulation up to T cell functions after days. These results are summarized in **Figure 6** as a percentage of signals compared to WT (100%; dashed line). Initial Ca^2+^ microdomains in the first milliseconds after TCR stimulation were reduced in *P2rx4* ^-/-^ (85%) and *P2rx7* ^-/-^ (54%) T cells (**Figures 1**, **6**), leading to a delayed global Ca^2+^ response in the cells lacking either P2X4 (16%) or P2X7 (19%) in the first minutes while the overall Ca^2+^ signal was not impaired. Still, the translocation of the nuclear factor NFAT-1 few minutes post-stimulation was significantly reduced (*P2rx4* ^-/-^ (71%) and *P2rx7* ^-/-^ (81%)) (**Figure 6**). Furthermore, T cell function is still impaired in *P2rx4* ^-/-^ and *P2rx7* ^-/-^ CD8^+^ T cells hours after stimulation resulting in impaired cytokine (IFN-γ: *P2rx4* ^-/-^ (52%) and *P2rx7* ^-/-^ (67%)) and granzyme B expression (*P2rx4* ^-/-^ (8%) and *P2rx7* ^-/-^ (26%)) as well as reduced proliferation of these cells after 3 days (*P2rx4* ^-/-^ (15%) and *P2rx7* ^-/-^ (25%)) (**Figures 5A–C**, **6**). Only in CD8^+^ T cells from *P2rx4* ^-/-^ mice, the expression of TNF-α was significantly reduced by 33%.

## 4 Discussion

In this study, we investigated the role of the purinergic cation channels P2X4 and P2X7 in the signal transduction of CD8^+^ T cells from very early cytosolic events to downstream responses. Upon TCR stimulation, Ca^2+^ microdomains are induced within milliseconds by the production of NAADP that binds to HN1L/JPT2 and engages RYR1, as well as the interaction of STIM1/2 with ORAI1 (3, 5, 6). Recently, we reported the influence of the purinergic channels P2X4 and P2X7 on the formation of initial Ca^2+^ microdomains in CD4^+^ T cells (21). In line with these results, Ca^2+^ microdomains were significantly reduced in the first millisecond after TCR stimulation in CD8^+^ T cells from *P2rx4^-/-^* and *P2rx7^-/-^* mice (**Figure 1**) or by addition of extracellular apyrase (**Figure 2**). Ca^2+^ microdomains are one of the first signaling events in a wide range of cell types, affecting different downstream pathways depending on the cell function and the source of the Ca^2+^ microdomains (35). While Ca^2+^ microdomains induced by the STIM-ORAI system are associated with ER Ca^2+^ refilling, PMCAs, and adenylyl cyclase activity, the activation of calcineurin, and the translocation of NFAT (36), lysosomal loading by ER-derived Ca^2+^ microdomains affects the endolysosomal system and dynamics (37). A similar fine-tuning mechanism for signal transduction represents the purinergic signaling system via P2X channels. While activation of the P2X1 channel via ATP concentrations in the nanomolar range maintains the mitochondrial metabolism (38), signaling through P2X4 regulates T cell migration (39), and the expression P2X7 is associated with the generation of memory cells following acute infections (40). Recently, it was shown that the ATP-releasing channel pannexin-1 promotes CD8^+^ T cell effector and memory responses through several intracellular pathways (41).

In the current study, we not only emphasized one aspect of T cell activity but tried to understand the whole cascade from initial to downstream responses. Thus, as the next step in the signaling cascade (after the formation of initial Ca^2+^ microdomains), we analyzed the global Ca^2+^ response in CD8^+^ T cells from *P2rx4^-/-^* and *P2rx7^-/-^* mice. The overall global Ca^2+^ response looked quite similar between WT and both knockout cells. The signal onset velocity was significantly reduced in T cells from *P2rx4^-/-^* mice, whereas T cells from *P2rx4^-/-^* and *P2rx7^-/-^* mice showed delayed responses to reach 200 nM Ca^2+^. CD4^+^ WT T cells respond to anti-CD3^+^ within approx. 115 s to reach 200 nM Ca^2+^ (20), whereas CD8^+^ WT T cells showed slower responsiveness of approx.140 s (**Figure 3C**). The delayed response observed in CD8^+^ T cells could be attributed to a higher activation threshold, which serves to avoid inappropriate activation and subsequent tissue damage resulting from their cytotoxicity (42). The strength and duration of a Ca^2+^ signal can shape the cellular response and determine between a certain proliferation program or cell death. Cytosolic and nuclear proteins translate the diverse Ca^2+^ signaling cascade into transcriptional activity (7).

One of these proteins is NFAT-1 (24). After dephosphorylation and translocation into the nucleus, NFAT-1 interacts with various other transcription factors and associated proteins, like activator protein 1 (AP1), forkhead box protein 3 (FOXP3), or Ikaros, in a stimulus-dependent manner. This cooperative DNA binding subsequently shapes immune cell response, like T cell differentiation or proliferation (43) and transcription of numerous genes, e.g. IFN-γ gene (IFNG) (28) and TNF-α (TNF) (31). Previous studies reported the impact of eATP and the P2X7 receptor on NFAT activation in Jurkat T cells and rat pheochromocytoma (PC12) cells (19, 44). Thus, we analyzed NFAT-1 translocation after TCR stimulation in CD8^+^ T cells from *P2rx4^-/-^* and *P2rx7^-/-^* mice (**Figure 2**). Strikingly, no or just a slight increase in NFAT-1 translocation was observed for T cells from *P2rx4^-/-^* and *P2rx7^-/-^* mice compared to the WT T cells after 7.5 min and 15 min of bead stimulation (**Figure 4B**). These results highlight the role of the purinergic channels P2X4 and P2X7 on the activation of CD8^+^ T cells by influencing not only the initial Ca^2+^ signals but also the translocation of the transcription factor NFAT-1.

Activated CD8^+^ T cells start to produce TNF-α and IFN-γ after TCR stimulation. We observed an increase in TNF-α expression of 17% of WT CD8^+^ T cells after 4h of stimulation and an increase IFN-γ expression of WT CD8^+^ T cells (20%) after 18 h of stimulation. The increase in CD8^+^ T cells expressing IFN-γ is regulated at the transcriptional level (45) and IFN-γ as well as TNF-α production is dependent on NFAT-1 (27, 31). The current study also highlights this relationship between NFAT-1 translocation and the production of pro-inflammatory cytokines. Both, NFAT-1 translocation minutes after TCR stimulation as well as CD8^+^ T cells expressing TNF-α and IFN-γ within hours of stimulation, were significantly reduced in T cells from *P2rx4^-/-^* and *P2rx7^-/-^* mice (**Figures 4**, **5**). The synthesis of granzyme B is tightly regulated at both transcriptional and translational levels and the transcriptional activation is induced by the stimulation of the T cell receptor as well as co-stimulation with cytokines (46). Furthermore, the release of granzyme B via granule exocytosis at the immunological synapse is dependent on the rises of free cytosolic Ca^2+^ (47). Hence, if the Ca^2+^ response is impaired in T cells from *P2rx4^-/-^* and *P2rx7^-/-^* mice this will lead to a decreased expression of granzyme B which is in line with our experiments (**Figure 5D**).

Moreover, TCR signaling is linked to the immediate expression of the NR4A family of nuclear orphan receptors and their transcripts can be used as biomarkers for TCR signaling (48). One member of the NR4A family is Nur77 (NR4A1). Nur77 is a negative transcriptional regulator of T cell activation, cell cycle progression, and proliferation that is upregulated within hours of TCR signaling (48–50). Therefore, Nur77 is an ideal tool for analyzing TCR signaling-dependent differences in the first hours after stimulation in our WT and knockout T cells. In line, we observed significantly reduced Nur77 expression in T cells from *P2rx4^-/-^* and *P2rx7^-/-^* mice 18h after stimulation. It is important to note that this only refers to TCR-induced Nur77 expression, as basal expression is unchanged in CD8^+^ T cells from WT mice compared to those from *P2rx4^-/-^* and *P2rx7^-/-^* mice.

T cell proliferation is regulated by various transcription factors, like NFAT, AP-1, NFκB, or Nur77 (45, 51, 52). We showed a significantly decreased proliferation in CD8^+^ T cells from *P2rx4* ^-/-^ and *P2rx7* ^-/-^ mice (**Figure 5C**). Previous reports indicate the influence of the duration and amplitude of Ca^2+^ signals on the activation of the pro-inflammatory transcriptional regulators NFAT, AP-1, NFκB (52). Further reports again highlighted the role of short-lived local Ca^2+^ signals on the translocation of NFAT-1 (53, 54). Here, we clearly demonstrate the association of Ca^2+^ signaling, transcription factor, and cytokine expression with the proliferation of CD8^+^ T cells. Impaired Ca^2+^ microdomain formation in *P2rx4* ^-/-^ and *P2rx7* ^-/-^ CD8^+^ T cells directly after T cell stimulation results in significantly delayed global Ca^2+^ signaling (**Figure 3C**), while the overall Ca^2+^ response was not changed (**Figure 3D**). Furthermore, in cells lacking either P2X4 or P2X7 channel NFAT-1 translocation several minutes after TCR stimulation and TNF-α, IFN-γ, Nur77 and granzyme B expression were impaired within hours of stimulation and, finally, slightly reduced proliferation of these cells after days was demonstrated (**Figure 6**). Previous work has discussed the importance of the duration and amplitude of a Ca^2+^ signal to the downstream response. While it is claimed that oscillation frequency influences gene transcription of certain transcription factors (52, 55) and NFAT-1 translocation depends on short-lived Ca^2+^ microdomains (53), the proliferation of cells is controlled by a full and global Ca^2+^ signal (56). This is in line with our data. In cells lacking either P2X4 or P2X7 channels, there are profound effects on Ca^2+^ microdomain formation, affecting NFAT-1 translocation as well as TNF-α and IFN-γ expression, whereas the global Ca^2+^ response is only slightly affected and, consequently, cell proliferation.

Although the affinities of P2RX4 and P2RX7 for eATP are different, with P2RX4 having a higher affinity (1-10 µM EC_50_) as P2RX7 (>100 µM EC_50_) for ATP (57), the knock-out of either receptor still had similar outcomes. The effects of P2RX4 were greatest on initial Ca^2+^ signals, which could be explained by the higher affinity for ATP especially during the first milliseconds to seconds after TCR stimulation, NFAT-1, which is known to be de-phosphorylated upon local Ca^2+^ rises, and TNF-α expression. So, in line small local rises in ATP trigger Ca^2+^ responses and NFAT translocation. In contrast, P2RX7 has stronger effects in IFN-γ and granzyme B expression as well as proliferation, which is at a time point (18h to 72h) where higher eATP concentrations are present due to release from activated immune cells (57) and/or dying of virus-infected cells (58). Previously we described that there is co-localization of P2RX4 and P2RX7 in CD4^+^ T cells at basal state which is significantly increased 5 mins after TCR stimulation (21) hinting to the possible formation of heterotrimers. The formation of heterotrimers in CD4^+^ T cells and hence possibly also in CD8^+^ T cells could explain these overall quite similar effects in T cells from *P2rx4^-/-^* and *P2rx7^-/-^* mice. If either of the two receptors is absent possibly no fully functional receptors are expressed in CD8^+^ T cells from *P2rx4^-/-^* and *P2rx7^-/-^* mice.

Taken together, our data suggest that purinergic signaling enhances the initial Ca^2+^ events during CD8^+^ T cell activation and plays an essential role in the refinement of the downstream effector function, providing advanced possibilities for clinical interventions, for example, in the tumor microenvironment.

## Data availability statement

The original contributions presented in the study are included in the article/**Supplementary Material**. Further inquiries can be directed to the corresponding author.

## Ethics statement

The animal study was approved by the regulatory committee (Hamburger Behörde für Gesundheit und Verbraucherschutz, Veterinärwesen/Lebensmittelsicherheit, ORG 941 and 1115) which included tissues of animal origin. The study was conducted in accordance with the local legislation and institutional requirements.

## Author contributions

VB: Conceptualization, Data curation, Validation, Visualization, Writing – original draft, Writing – review & editing, Formal analysis, Investigation, Methodology. NL: Formal analysis, Investigation, Writing – review & editing. FM: Formal analysis, Investigation, Visualization, Writing – review & editing. MB: Formal analysis, Investigation, Writing – review & editing. TS: Formal analysis, Investigation, Writing – review & editing. L-MW: Methodology, Software, Writing – review & editing. MJ: Investigation, Writing – review & editing. JH: Supervision, Project administration, Funding acquisition, Writing – review & editing. FK-N: Funding acquisition, Project administration, Supervision, Writing – review & editing. BR: Funding acquisition, Project administration, Supervision, Writing – review & editing. H-WM: Funding acquisition, Project administration, Supervision, Writing – review & editing. AG: Conceptualization, Funding acquisition, Project administration, Supervision, Writing – review & editing. RW: Funding acquisition, Project administration, Software, Supervision, Writing – review & editing. B-PD: Conceptualization, Data curation, Funding acquisition, Project administration, Resources, Supervision, Validation, Visualization, Writing – original draft, Writing – review & editing.
