# Supplementary material for: Time-resolved role of P2X4 and P2X7 during CD8+ T cell activation
Source: Front Immunol. 2024 Feb 15;15:1258119. doi: 10.3389/fimmu.2024.1258119 (PMC10902106; doi:10.3389/fimmu.2024.1258119)
Supplement: Supplementary file 1 [file DataSheet_1.docx]

Supplementary Material

Time-resolved role of P2X4 and P2X7 during CD8+ T cell activation

Valerie J. Brock^1^, Niels Christian Lory^2^, Franziska Möckl^1^, Melina Birus^2^,Tobias Stähler^2^, Lena-Marie Woelk^3,4^, Michelle Jaeckstein^5^, Joerg Heeren^5^, Friedrich Koch-Nolte^2^, Björn Rissiek^6^, Hans-Willi Mittrücker^2^, Andreas H. Guse^1^, René Werner^3,4^, Björn-Philipp Diercks^1^*

^1^The Calcium Signalling Group, Department of Biochemistry and Molecular Cell Biology, University Medical Centre Hamburg-Eppendorf, Hamburg, Germany

^2^Department of Immunology, University Medical Centre Hamburg-Eppendorf, Hamburg, Germany

^3^Department of Applied Medical Informatics, University Medical Centre Hamburg-Eppendorf, Hamburg, Germany

^4^Department of Computational Neuroscience, University Medical Centre Hamburg-Eppendorf, 20246 Hamburg, Germany

^5^Department of Biochemistry and Molecular Cell Biology, University Medical Centre Hamburg-Eppendorf, Hamburg, Germany

^6^Department of Neurology, University Medical Centre Hamburg-Eppendorf, Hamburg, Germany

*** Correspondence:**Björn-Philipp Diercks
[b.diercks@uke.de](mailto:b.diercks@uke.de)

**
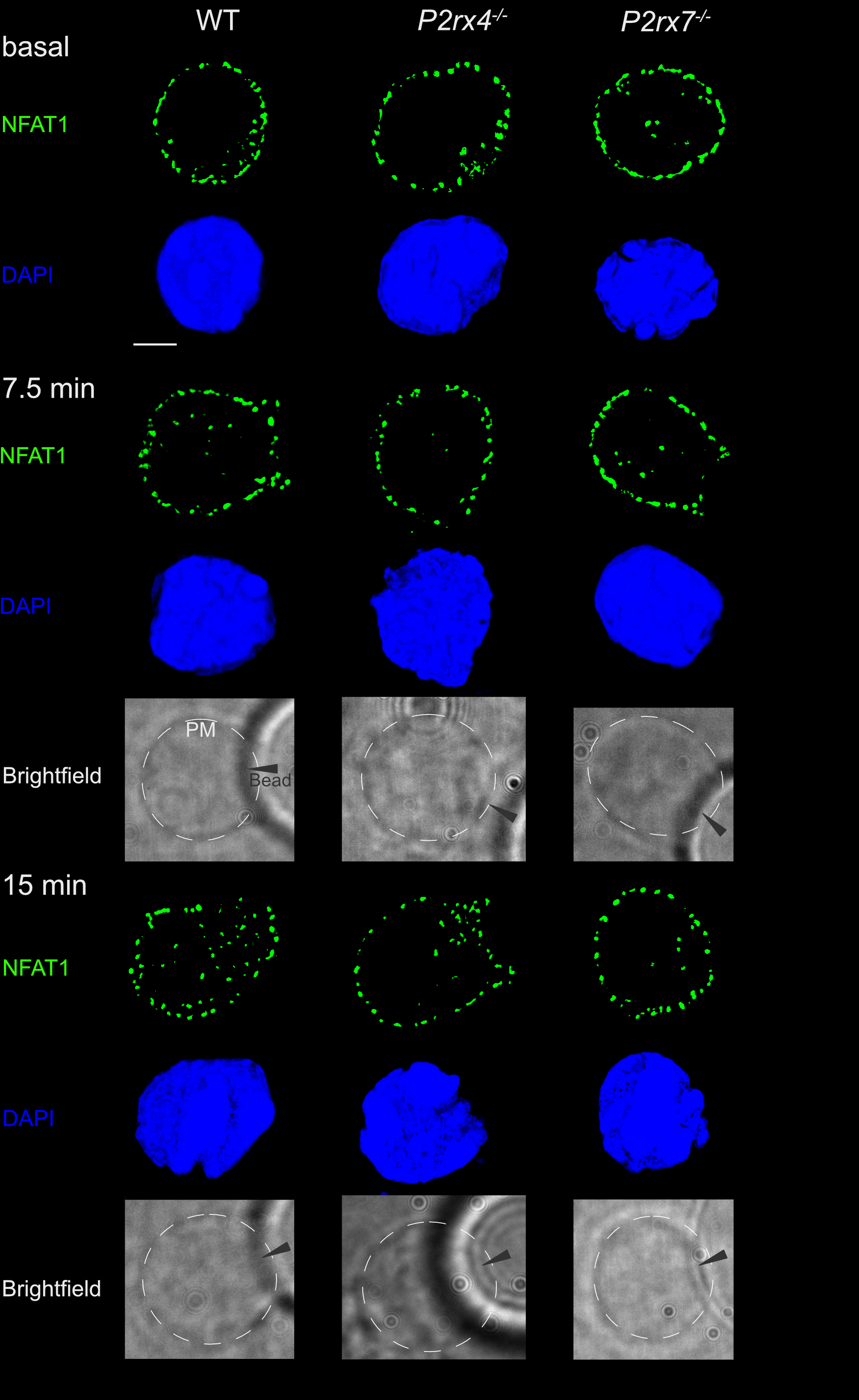
**

**Supplementary Figure 1: NFAT-1 is influenced by P2X4 and P2X7 minutes after TCR stimulation.**

Separate individual channels of the cells shown in Fig. 3 are shown for the time points indicated. NFAT-1 is shown in green, the cell nucleus in blue. The bead contact is shown schematically with black arrowheads and can be seen in bright field images. The white dashed circles show the outlines of the T cells.

**
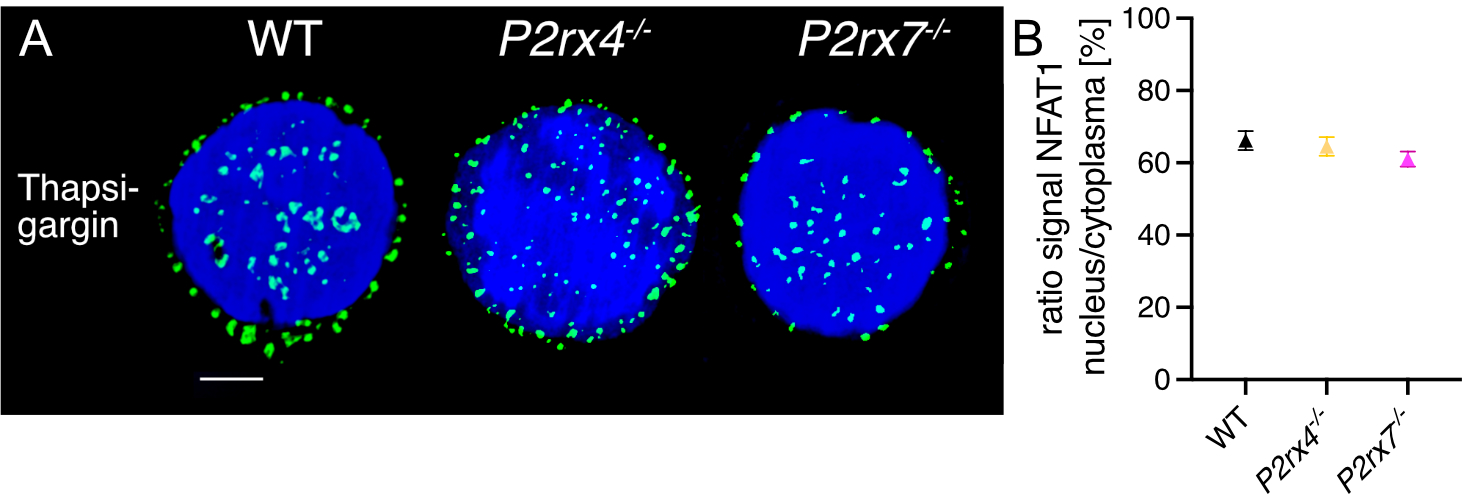
**

**Supplementary Figure 2: Thapsigargin response in CD8^+^ T cells from WT, *P2rx4^-/-^* and *P2rx7^-/-^* mice.**

CD8^+^ T cells were stimulated with 1.67 µM thapsigargin (Merck, Germany) for 5 min and NFAT-1 and the nucleus were stained as described. The thapsigargin response is comparable in WT, *P2rx4^-/-^* and *P2rx7^-/-^* cells. Data are mean ± SEM. WT n = 41, *P2rx4^-/-^* = 39 and *P2rx7^-/-^* = 41. (A) Characteristic CD8^+^ T cells from WT, *P2rx4^-/-^* and *P2rx7^-/-^* mice. Scalebar 2 µm. (B) Quantification of the NFAT-1 signal by the Ratio signal of NFAT-1 in the nucleus and the cytoplasm in percentage. Data were compared using ordinary one-way ANOVA and Tukey’s multiple comparisons test.

**Supplementary Figure 3: Expression analysis of the early surface marker CD69 and the transcription factor IRF4 in CD8^+^ T cells from WT, *P2rx4^-/-^* and *P2rx7^-/-^* mice.**

Spleen cells from WT, *P2xr4*^-/-^ and *P2xr7*^-/-^ mice were incubated with and without anti-CD3 mAb and anti-CD28 mAb. After 4h and 18h, expression of CD69 (A) and IRF4 (B) is given as mean fluorescence intensity (MFI), which was analyzed by FACS and of antibody staining of CD8^+^ T cells. Mean ± SEM, N = 3 per group. Results were analyzed with two-way ANOVA and Tukey’s post-test.


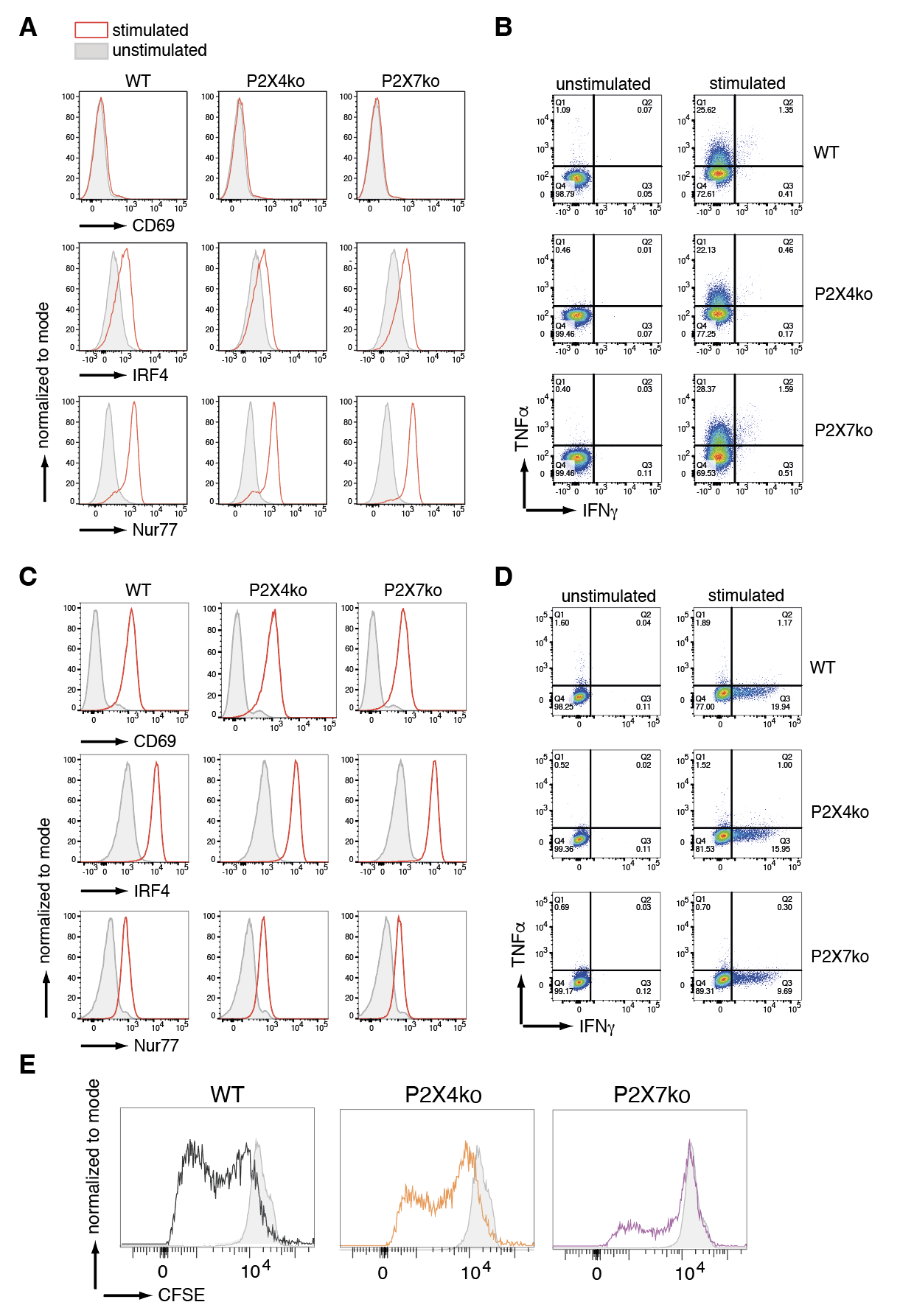


**Supplementary Figure 4: Representative histograms and dot plots for the expression of different markers after stimulation CD8^+^ T cells.**

A-D) Representative histograms for CD69, IRF4 and Nur77 staining (A,C) and dot plots for IFN-γ and TNF-α staining (B,D) of CD8^+^ T cells from WT, *P2rx4*^-/-^ and *P2rx7*^-/-^ mice cultured with (red lines) and without (grey) stimulation with anti-CD3 mAb and anti-CD28 mAb for 4h (A,B) or 18h (B,D). Data correspond to Figure 5A-D. E) Representative histograms for CFSE of CD8^+^ T cells from WT, *P2rx4*^-/-^ and *P2rx7*^-/-^ mice cultured with (lines) and without (grey) stimulation with anti-CD3 mAb for 72h. Data correspond to Figure 5F.


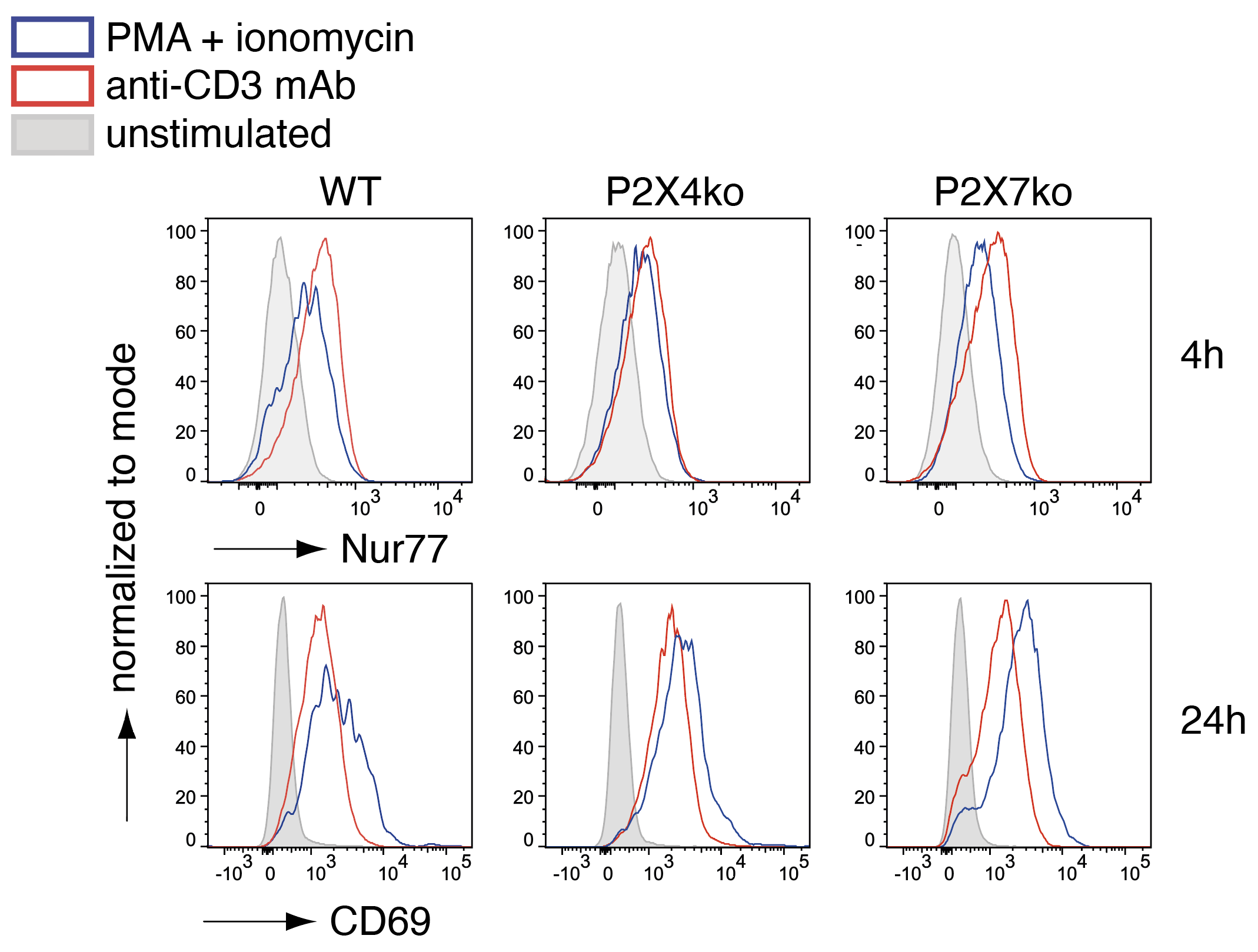


**Supplementary Figure 5: Representative histograms for CD8^+^ T cells stimulated with either anti-CD3 mAb or PMA and ionomycin.**

Representative histograms for Nur77 and CD69 staining of CD8^+^ T cells from WT, *P2rx4*^-/-^ and *P2rx7*^‑/-^ mice cultured with out stimulation (grey), with ant-CD3 mAb (red line) or with PMA and ionomycin (blue line) for 4h (IRF4) or 24h (CD69).
